# Development of Optimal Medium Content for Bioelements Accumulation in *Bacopa monnieri* (L.) In Vitro Culture

**DOI:** 10.1007/s12010-014-1095-8

**Published:** 2014-08-15

**Authors:** Maciej Łojewski, Bożena Muszyńska, Agata Smalec, Witold Reczyński, Włodzimierz Opoka, Katarzyna Sułkowska-Ziaja

**Affiliations:** 1 Department of Pharmaceutical Botany, Jagiellonian University Collegium Medicum, ul. Medyczna 9, 30-688 Kraków, Poland; 2 Faculty of Materials Science and Ceramics, AGH University of Science and Technology, Kraków, Poland; 3 Department of Inorganic and Analytical Chemistry, Jagiellonian University Collegium Medicum, ul. Medyczna 9, 30-688 Kraków, Poland

**Keywords:** AAS, Anthranilic acid, *Bacopa monnieri*, Bioelements, l-tryptophan

## Abstract

*Bacopa monnieri* is one of the most interesting plants from the Ayurveda system. The aims of present research were, basing on in vitro shoot culture of *B. monnieri*, to determine content and to evaluate the influence of physiologically important metabolites on the selected bioelements accumulation in biomass. The most significant increase in biomass production was observed in the culture medium enriched with 0.5 mg/L of anthranilic acid. In this medium also, the highest accumulation of Mg was noted. The highest concentration of iron was determined in *B. monnieri* in vitro culture enriched with 0.25 g/L of serine. The addition of l-tryptophan, magnesium sulfate, and zinc hydroaspartate caused only a small increase in the accumulation of copper in *B. monnieri*. Increase in Zn accumulation was obtained in biomass from in vitro culture of *B. monnieri* with the addition of magnesium sulfate and zinc hydroaspartate. In the case of Na, the maximum level of this element was in biomass from medium enriched with zinc hydroaspartate. Twofold increase in K concentration was obtained in biomass from cultures on medium with addition of serine and magnesium sulfate. The concentrations of Ca in biomass of all studied media were at the similar level.

## Introduction

*Bacopa monnieri* (L.) Pennell (water hyssop), known locally in India as Brahmi or Jalanimba, is one of the most important plants in the traditional Hindu system of medicine, Ayurveda. Brahmi name comes from the word Brahma, one of the main gods of Hinduism. *B. monnieri* is used in India for 5,000 years to treat epilepsy and insomnia, as a sedative and abolishing raw anxiety. Indian materia medica (Bhavprakasha Nighantu 1,500 years AD) recommends this resource as a means of improving memory and concentration [1–5]. Commercially available preparations of *B. monnieri* improve brain function and improve concentration and memory in both young and older people [6]. Clinical studies confirm beneficial effects of this species in recovering of mental functions in children suffering from ADHD, improving the cognitive functions in patients after stroke and epilepsy [7–10]. Based on the previous studies, it is believed that these effects are due to the ability to modulate the cholinergic system of the plant extracts [11]. Their main action is to increase blood flow in the brain, improving concentration, they also have antioxidant, anti-inflammatory, antibacterial, and antitumor effects, and they are also used as support in neurodegenerative diseases (such as Alzheimer’s and Parkinson’s diseases) treatment [12–25]. Based on studies of patients with depression, it was shown that *B. monnieri* exhibits an antidepressant activity [1, 11]. Compounds, which are attributed to the abovementioned actions, are bacosides and triterpenoids belonging to the steroid saponins [1].

Extracts from *B. monnieri* are used for blood purification from heavy metals, due to their ability to accumulate organic compounds contained therein [3]. This raises the possibility of obtaining raw material enriched in beneficial to human body compounds (anthranilic acid-L_1_ vitamin; l-tryptophan; serine) and micronutrients (Mg and Zn) for targeted physiological effects. The fresh material might be even more effective in treatment of diseases. Due to limited information on the content of macroelement and microelement (playing important role in human metabolism by building blocks and being enzymes activators) in the fresh material of *B. monnieri*, it is necessary to determine bioelements quantitatively and to assess their ability to accumulate in this material under fully controlled conditions of in vitro culture. To study the mechanism of bioelements accumulation in shoots of *B. monnieri*, it was necessary to develop an appropriate in vitro culture medium composition and culture conditions.

The aim of present research was to evaluate the influence of the necessary amino acids for the human body (anthranilic acid, l-tryptophan, serine) on accumulation of the selected bioelements. Anthranilic acid (L_1_ vitamin) and serine are precursors of l-tryptophan, which in turn is a direct precursor of serotonin. l-tryptophan when ingested, on the contrary to serotonin, easily crosses the blood–brain barrier in the central nervous system, where it is efficiently converted to serotonin. In the central nervous system, serotonin takes part in neuron to neuron communication and appears to enhance the perception of well-being and modulate the intensity of emotional states. Serotonin has some cognitive functions, including antidepressant, enhancing memory, and learning [26, 27]. Mg and Zn have antidepressant activity, and Cu, Fe, K, Na, and Ca influence homeostasis in the human body. So, the next aim of the current work was to create composition of medium which guarantees the optimal increase of biomass, optimal concentration of chosen organic compounds which lead to maximal accumulation of the selected physiologically active elements.

To obtain samples (biomass from in vitro cultures) with expected amount of the chosen elements, culture media composition was modified, in vitro culture increments were constantly observed, and the elements were quantitatively analyzed by atomic absorption spectroscopy (AAS) and atomic emission spectroscopy (AES) methods. This work has interdisciplinary character connecting photochemistry, biotechnology, and physiology of higher plants.

## Experimental

### Chemicals

All used chemicals were of analytical grade: MgSO_4_ was purchased from POCh (Gliwice, Poland); zinc hydroaspartate was from Farmapol (Poland); conc. HNO_3_ and H_2_O_2_ suprapure were from Merck (Darmstad, Germany); and anthranilic acid, l-tryptophan, and serine (purity ≥98 % by HPLC) were from Sigma-Aldrich (St. Louis, USA). The growth regulators naphthalene-1-acetic acid (NAA) and 6-benzylaminopurine (BAP) were also from Sigma-Aldrich. Water was filtered through Millipore Millex-GP, 0.22 μm, and was purified by quadruple distillation.

### Methods

#### Initial Cultures

The studies were conducted on in vitro shoot cultures of *B. monnieri*. The in vitro cultures were established from commercially available *B. monnieri* already placed in vitro from IVPLANT Company (representative samples of *B. monnieri* in vitro cultures were deposited at the Department of Pharmaceutical Botany, Jagiellonian University Collegium Medicum, Kraków, Poland). The initial biomass amounted to 4.0 g/250 mL of medium. The shoots (stems, with leaves) were cut into small pieces and transferred to Erlenmeyer flasks and cultured on liquid Murashige and Skoog (MS) medium (250 mL) [28] with nicotinic acid, myo-inositol and vitamin B_1_ (4,0 mL/L), and growth regulators BAP 1.0 mg/L and NAA 0.2 mg/L according to Chaplot Binita [29] with our modification. This was the basal medium, and shoots were subcultured every 4 weeks.

### Experimental In Vitro Cultures

Shoots from basal medium were used to obtain culture in vitro on modified medium. This was the basal medium to which the following reagents were added: 0.1 g/L Mg (magnesium sulfate), 0.1 g/L Zn (zinc hydroaspartate), 0.1 g/L l-tryptophan, 0.25 g/L and 0.5 g/L serine, and 0.5 mg/L anthranilic acid. For metals, the assignment of indicator values is more complex. Metal can exist in the environment as an element or as a compound with other inorganic or organic elements (chelates) [30]. Applied concentration of amino acids and elements was added to the media based on the optimum concentrations experimentally developed of their levels for use in plant culture media [28]. Cultures were grown under constant artificial light (4 W/m^2^, LF-40W lamp, daylight, Piła) at 25 ± 2 °C. The cultures were shaken at a rate of 140 rpm (shaker Altel, Poland). After 4 weeks, the fresh biomass was collected, frozen, and lyophilized (lyophilizer Freezone 4.5, Labconco; temperature −40 °C). After lyophilization, plant material was weighed (5 g of each) and grounded in an agate mortar. Then, the powdered dry mass was used for quantitative elemental analysis by atomic absorption spectroscopy.

### Quantitative Determination of Elements in Biomass from In Vitro Cultures of *B. monnieri*

Prior to the elements quantitative analysis, plant material was wet digested (conc. HNO_3_ and H_2_O_2_) in a microwave system (Multiwave 3000, Anton Paar, Switzerland). Concentrations of the elements were determined by means of atomic absorption spectrometry using flame technique (Mg, Fe, Zn, Cu, and Ca) and by means of flame photometry method (K, Na). PerkinElmer atomic absorption spectrometer Model 3110 (USA) was used in all measurements. Each sample was analyzed in quadruplicate, and the results presented below are the mean values.

The accuracy and precision of the measurements were tested with the use of the Certified Reference Material, Mixed Polish Herbs No INCT-MPH-2. Satisfactory agreement between the determined and certified elements concentration values was achieved.

### Statistical Analysis

The statistical analyses were performed using Student’s *t* test. For each of the obtained materials of *B. monnieri* from in vitro cultures, 12 samples were used for the determination of each compound and all the analyses were carried out in four repetitions. The results were expressed as mean values and standard deviation (SD). All the calculations were conducted using Statistica 10 (StatSoft, Poland) and Statgraphics Centurion XVI (Poland). Statistical significance was defined at *p* < 0.05.

## Results and Discussion

Fresh biomass of in vitro shoot culture of *B. monnieri* increased 14-fold during 4-week growth cycles on the tested variants of MS medium with nicotinic acid, myo-inositol and vitamin B_1_ (4.0 mL/L), and growth regulators BAP 1.0 mg/L and NAA 0.2 mg/L. Fourteenfold biomass increments were also observed on the medium variants containing anthranilic acid, and the similar (above 13-fold) increments were obtained on medium with addition of serine and magnesium sulfate. The lower biomass increments were obtained on the medium containing 0.1 g/L l-tryptophan (9-fold) and the lowest in the case of the medium with addition of 0.1 g/L Zn (zinc hydroaspartate) (8-fold).

Nevertheless, increases in biomass were satisfactory, as the average biomass increase obtained by other authors was at similar level [31, 32].

As a part of the presented research, concentrations of the selected biologically active bioelements (Mg, Zn, Cu, Fe, Ca, K, and Na) in biomass of in vitro culture of *B. monnieri* were examined. Concentrations of the elements in the plant material cultured on media enriched with those elements (Mg and Zn) were also determined. The obtained results illustrate the influence of chosen organic compounds (anthranilic acid, l-tryptophan, serine) on accumulation of bioelements in in vitro shoot cultures of *B. monnieri*. The largest increase in biomass and simultaneously the highest accumulation of Mg were obtained in the case of MS medium enriched with anthranilic acid.

**Fig. 1:**
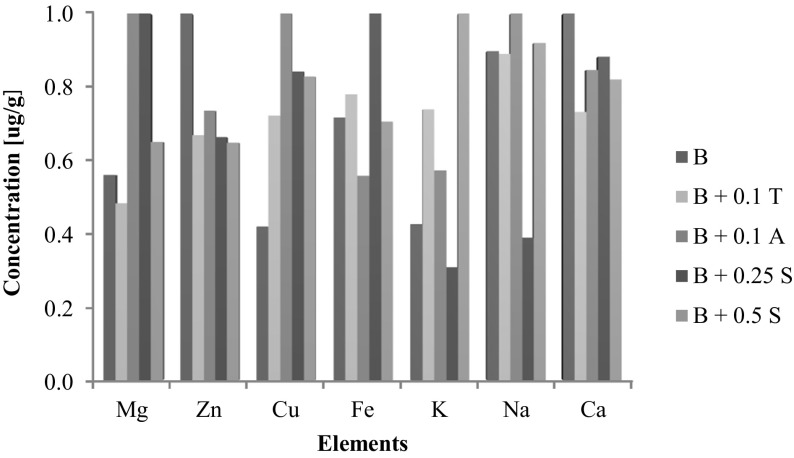
The dependence of the accumulation rate (normalized concentrations) of the elements in in vitro cultures of *Bacopa monnieri* (*A* anthranilic acid, *B* shoots of *B. monnieri* from in vitro culture, *S* serine, *T* l-tryptophan)

The results of quantitative analysis of individual elements in the ongoing culture allowed defining the relationships between the elements accumulation rate and the conditions of in vitro culture (culture medium composition). Considering the weight gains in in vitro culture of *B. monnieri*, it can be concluded that they are comparable to the control culture or lower in the case of culture with addition of zinc and tryptophan. What is more important, the developed in vitro cultures are characterized by high weight gain and relatively high capacity for accumulation of the bioelements. It is clearly visible on the three-dimensional Fig. 1 which has been drawn up based on the results of the analysis summarized in Table 1. Basing on Fig. 1 and Table 1, it was discussed to what extent enrichment of in vitro culture media of *B. monnieri* with particular substances (anthranilic acid, serine, l-tryptophan, and magnesium salts and zinc) influences the elements accumulation in the plant.

**Table 1:** Concentrations of the elements in in vitro culture of *B. monnieri* (μg/g dry weight)

Sample	Weight gains/increase biomass	Mg	Zn	Cu (μg/g DW)	Fe	K	Na	Ca
B	14	737.8 ± 1.5**	218.1 ± 7.0*	1.6 ± 2.2	203.2 ± 2.8*	19,300.0 ± 0.8**	555.2 ± 10.2*	2,894.5 ± 1.1**
B 1 + 0.1 Mg	12	971.3 ± 0.3**	274.4 ± 6.9*	3.4 ± 2.8	161.5 ± 1.1**	45,887.5 ± 1.0**	713.9 ± 0.5**	2,487.3 ± 0.5**
B 2 + 0.1 Mg		955.3 ± 1.7**	311.0 ± 7.7*	2.6 ± 2.9	171.1 ± 0.8**	42,970.0 ± 12.9**	734.3 ± 0.4**	2,917.3 ± 0.4**
B 1 + 0.1 Zn	8	570.8 ± 2.2**	795.1 ± 0.6**	4.1 ± 1.1	266.2 ± 1.1**	19,102.5 ± 1.2**	822.6 ± 3.8**	2,078.3 ± 0.8**
B 2 + 0.1 Zn		496.5 ± 0.9**	870.7 ± 1.2**	2.8 ± 2.2	239.7 ± 0.6**	20,802.5 ± 3.7**	926.5 ± 2.0**	2,027.8 ± 1.8**
B 1 + 0.1 T	9	571.3 ± 1.2**	135.9 ± 1.9*	2.7 ± 3.3	234.5 ± 1.5**	34,157.5 ± 0.8**	563.7 ± 2.2**	2,165.0 ± 0.9**
B 2 + 0.1 T		706.3 ± 3.4**	155.6 ± 3.0*	2.8 ± 5.0	206.7 ± 2.9*	32,717.5 ± 2.9**	537.6 ± 2.4**	2,082.0 ± 2.7**
B 1 + 0.5 A	14	1,537.5 ± 1.0**	163.1 ± 1.7*	3.7 ± 1.8	175.0 ± 1.3**	24,830.0 ± 6.9**	591.9 ± 5.0**	2,335.5 ± 1.4**
B 2 + 0.5 A		1,097.5 ± 1.6**	158.3 ± 2.9*	3.9 ± 0.8	140.5 ± 1.4**	26,870.0 ± 3.4**	645.3 ± 2.4**	2,568.8 ± 0.7**
B1 + 0.25 S	13	963.3 ± 3.8**	161.9 ± 1.9*	3.2 ± 9.1	250.3 ± 9.1*	14,692.5 ± 0.5**	239.2 ± 4.4*	2,447.8 ± 1.6**
B2 + 0.25 S		1,087.5 ± 1.4**	127.3 ± 1.1**	3.2 ± 4.2	315.1 ± 4.2*	13,215.0 ± 0.2**	243.4 ± 2.7*	2,662.8 ± 1.2**
B 1 + 0.5 S	13	836.8 ± 1.3**	147.7 ± 1.4**	3.4 ± 0.9	224.8 ± 0.7**	44,097.5 ± 4.3**	552.5 ± 7.1*	2,355.5 ± 1.4**
B 1 + 0.5 S		874.0 ± 3.0**	134.5 ± 3.7*	2.9 ± 2.2	173.6 ± 0.8**	46,227.5 ± 1.9**	586.1 ± 8.8*	2,397.5 ± 5.8**

Data were presented as the mean ± SD; *n* = 4 repetitions. *p < 0.05, **p < 0.01 by Statistica 10 (StatSoft, Poland)

*B* shoot in vitro culture from MS medium (control), *B 1 + 0.1 Mg* and *B 2 + 0.1 Mg* in vitro culture from MS medium with addition of 0.1 g/L MgSO_4_, *B 1 + 0.1 Zn* and *B 2 + 0.1 Zn* in vitro culture from MS medium with addition of 0.1 g/L Zn hydroaspartate, *B 1 + 0.1 T* and *B 2 + 0.1 T* in vitro culture from MS medium with addition of 0.1 g/L L-tryptophan, *B 1 + 0.5 A* and *B 2 + 0.5 A* in vitro culture from MS medium with addition of 0.5 mg/L anthranilic acid, *B1 + 0.25 S* and *B2 + 0.25 S* in vitro culture from MS medium with addition of 0.25 g/L serine, *B 1 + 0.5 S* and *B 1 + 0.5 S* in vitro culture from MS medium with addition of 0.5 g/L serine

Plant species differ substantially in all aspects connected with mineral plant nutrition: ion uptake, transport, and accumulation. Mechanisms involved consist of genetic features, environmental conditions, ion properties, and transport paths. Genetics is the base of plant diversity; environmental conditions are the same plant species that can or cannot grow in environment conditions characterized by drastically variable elements concentrations; ion properties are valency, speciation, and concentrations that influence element uptake and transport in plants; and transport is for example divalent cations (Zn, Cd, Hg, Cu, Pb) that influence the trans-root potential [30]. Studies presented that Cu and Zn interact with each other due to antagonistic relationship, but Mg and organic compounds increased the level of Cu in plants [30, 33]. Cu concentration (Table 1, Fig. 1) in in vitro culture of *B. monnieri* was in the range of 1.6–4.1 μg/g dry weight (DW). The addition of Zn, l-tryptophan, Mg, or serine caused only a small increase in the accumulation of copper in *B. monnieri*. This effect was most noticeable with the addition of 0.5 mg/L anthranilic acid and 0.25 and 0.5 g/L of serine to culture medium. The addition of these compounds caused double increase in bioaccumulation of Cu in relation to the culture on liquid medium without additives. This increase was up to 3.9 μg/g DW for the addition of 0.5 mg/L of anthranilic acid, 3.2 and 2.9–3.4 μg/g DW in the case of additions of 0.25 and 0.5 g/L of serine, respectively. Copper concentration in the obtained material is comparable to that of legumes [34].

*B. monnieri* in vitro cultures enriched with magnesium sulfate (0.1 g/L) were characterized by a slight increase in this element accumulation which concentration ranged from 955.3 to 971.3 μg/g DW. Similar relationship was observed in the case of cultures enriched with 0.25 and 0.5 g/L of serine–Mg concentration that was between 836.8 and 1,087.5 μg/g DW. The addition of zinc hydroaspartate (0.1 g/L) and l-tryptophan (0.1 g/L) to the cultures resulted in decrease of Mg accumulation in biomass, which in the first case was in the range from 496.5 to 570.8 μg/g DW while in the second 571.3–706.3 μg/g DW. It has been found that addition of 0.5 mg/L of anthranilic acid caused the greatest increase in magnesium accumulation in in vitro culture of *B. monnieri* and was in the range 1,097.5–1,537.5 μg/g DW. Mg concentration in the material is high and comparable with the best sources of this nutrient like cocoa or buckwheat [34].

Zinc in biomass from in vitro culture of *B. monnieri* was at the level of 134.5–870.7 μg/g DW. No increase of zinc accumulation was observed after addition of Mg, l-tryptophan, serine, or anthranilic acid. The enrichment of the culture medium with 0.1 g/L of zinc hydroaspartate increased its bioaccumulation four times (up to 870.7 μg/g DW) with respect to *B. monnieri* grown on liquid medium without supplements, in which Zn concentration was 218.1 μg/g DW. Other studies showed synergism between Zn and Fe, while Mg content was not affected by Zn levels [35]. The concentration of Zn in *B. monnieri* is higher than in the best dietary source of this the element—wheat germ (150 μg/g DW) [34].

The concentration of Fe in the biomass obtained from in vitro culture of *B. monnieri* on liquid medium without additives was equal to 203.2 μg/g DW. In this case, the enrichment of culture with Mg, Zn, l-tryptophan, serine, or anthranilic acid did not cause substantial increase of iron accumulation in the plant material. Iron concentration was in the range 140.5–315.1 μg/g DW and was the highest in culture enriched with the addition of 0.25 g/L serine. Nevertheless, iron concentration in the raw material is relatively high and comparable to the element level in vegetables and in edible mushrooms [34, 36].

The concentration of potassium in in vitro culture of *B. monnieri* with no additives was at the level of 19,300.0 μg/g DW. In the case of culture with 0.1 g/L magnesium sulfate addition, double increase in accumulation of potassium was observed (K concentration range 42,970.0–45,887.5 μg/g DW). Similar phenomenon of potassium higher accumulation was observed for cultures containing 0.5 mg/L of serine (K concentration range 44,097.5–46,227.5 μg/g DW). In other cases, there was no significant increase in the element bioaccumulation. Very high levels of potassium in the studied biomass were approximately four times higher than those in dried apricots and figs (one of the best source of potassium) [34].

The sodium concentration in in vitro shoots culture of *B. monnieri* was in the range of 239.2–926.5 μg/g DW. It was observed that the addition of serine or l-tryptophan did not significantly increase accumulation of sodium. The addition of 0.25 g/L serine reduced sodium concentration by half. In the case of anthranilic acid addition, no accumulation of Na was noted. Increased sodium absorption effect was most noticeable in the case of cultures enriched with 0.1 g/L zinc hydroaspartate and 0.1 g/L magnesium sulfate. The sodium content was 713.9–734.3 μg/g DW in the case of magnesium sulfate addition and 822.6–926.5 μg/g DW for zinc addition. Analysis of calcium concentration in the culture with the enriched media clearly showed no visible growth in bioaccumulation of this element. The concentration of calcium in biomass of in vitro culture of *B. monnieri* was in the range of 2,027.8–2,917.3 μg/g DW. Anyway, Ca concentration in cultured fungi material is higher than that in its best source, i.e., walnuts [34].

### Chemometric Analysis and Cluster Analysis

In order to conduct more precise data analysis, chemometric methods were used. One of the useful chemometric tools is cluster analysis [36–40]. This method allows distinguishing, out of the complete set of objects, groups of similar objects characterized by more than one feature. Cluster analysis (CA) belongs to unsupervised learning methods. This means that the analyst prior to the analysis did not have information on the classification of objects. The analyzed objects are recognized similar when their location in multidimensional space is imminent. The result of the analysis is distribution into groups of objects having a high level of similarity.

**Fig. 2:**
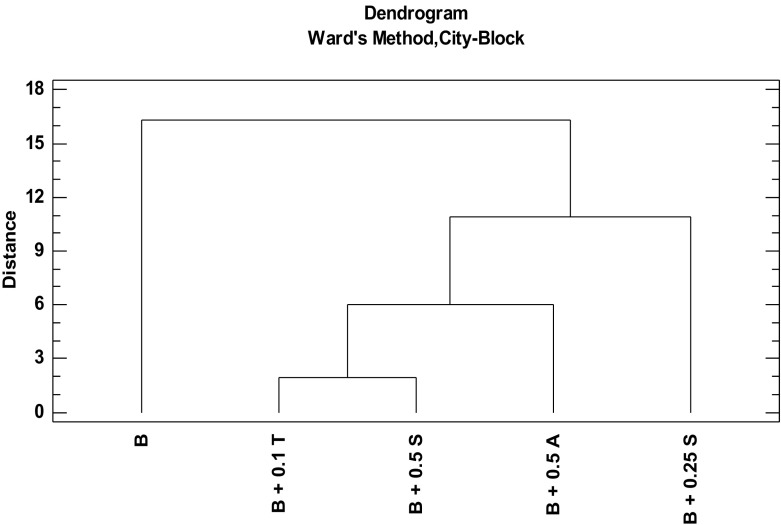
Dendrogram presenting similarity of objects (various versions of in vitro culture media; the city block distance, Ward’s algorithm). In vitro shoot culture from MS medium (control) (*B*). In vitro culture from MS medium with addition of 0.1 g/L l-tryptophan (*B + 0.1 T*). In vitro culture from MS medium with addition of 0.5 mg/L anthranilic acid (*B + 0.5 A*). In vitro culture from MS medium with addition of 0.25 g/L serine (*B + 0.25 S*). In vitro culture from MS medium with addition of 0.5 g/L serine (*B + 0.5 S*). Data were presented as the mean ± SD; *n* = 4 repetitions. *p < 0.05, **p < 0.01 by Statistica 10 (StatSoft, Poland)

**Fig. 3:**
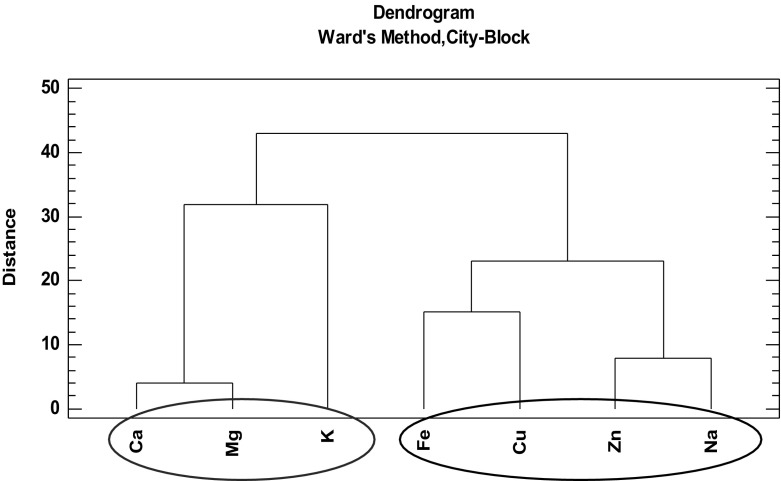
Dendrogram presenting similarity of changes in bioelements concentrations in the biomass of *B. monnieri*

The results of similarity analysis are presented in graphic form of the so-called dendrograms. The x-axis and y-axis labels do not correspond to the numerical axis (Figs. 2 and 3). On the dendrogram, x-axis corresponds to the name of the object to be analyzed, while y-axis corresponds to the distance between the objects. In this work, the distance between objects is defined as a city block. Application of Ward’s agglomeration method, which is based on the concept of analysis of variance, allowed defining the distance between clusters.

It can be concluded that modifications of in vitro culture medium lead to substantial changes in bioelements accumulation in the analyzed plant material (Fig. 2) as non-modified medium is completely different from the other media (in respect to its effectiveness to promote bioelements accumulation in *B. monnieri* biomass). Definitely, similar results were observed for media containing l-tryptophan and higher concentration of serine—these elements formulate a distinct cluster. Addition of anthranilic acid gives similar results (close position in the discussed dendrogram). Strangely enough, it was found that addition of lower amount of serine (0.25 g/L of culture medium) leads to different bioelements accumulation than when serine addition is higher (0.5 g/L).

Through the analysis of the element similarity (bearing in mind similarity of the course of elements concentration changes, not the absolute elements concentration values), two clusters can be distinguished: first one consisting of macroelements, i.e., K, Mg, and Ca; and the second one consisting of microelements, i.e., Fe, Cu, Zn, and Na. Most probably, these results from elements accumulation mechanisms are involved. K, Mg, and Ca are taken up actively by the plants against the element chemical activity gradient in the tissue and medium. The fact that microelements behave similarly means that the elements form a distinct cluster, first cluster (Ca, Mg, K) and second cluster (Cu, Zn, Na, Fe), suggest more direct influence of medium composition on transport mechanism. The higher is concentration of the element in culture medium, the higher is its accumulation in biomass, for example, concentration of magnesium in shoot in vitro culture from medium with no additives is 937.8 μg/g DW in comparison to in vitro culture from medium with addition of 0.1 g/L magnesium sulfate, concentration of magnesium is 971.3 μg/g DW.

### Principal Component Analysis

The second chemometric analysis methods used in the work are the principal component analysis, which is a calculation method allowing the reduction of variables [35, 38, 40]. Reducing the amount of variables involves changing the initial set of variables into the new, reduced in number set of the so-called principal components.

In the discussed topic, the basic set of variables was formulated by the elements concentration changes attributed to a given in vitro culture medium composition (seven variables). After PCA analysis, the new set of principal components (C_1_, C_2_, C_3_) turned out to be good enough to describe 81.7 % of the determined variability of objects (culture media). The new variables C_1_, C_2_, C_3_ resulted from linear combination of input variables, which are multiplied by the corresponding loads. The load is adequate with saturation of the variable specified factor.

Table 2 shows the collected values and the individual loads for each of the three major components. Presentation of the results in this form allows quick and easy interpretation of them and determination which variables have a significant impact on the main components C_1_, C_2_, and C_3_.

**Table 2:** The values of three main component weights in relation to the determined elements

Elements	Component 1	Component 2	Component 3
Ca	0.450987	−0.0146789	−0.309559
Cu	−0.097783	−0.0232271	0.814127
Fe	−0.227619	0.62657	−0.0527515
K	0.125636	−0.595109	−0.0847377
Mg	0.465107	−0.0151984	0.480648
Na	−0.414253	−0.500899	−0.00982871
Zn	−0.575781	−0.0370028	0.0169702

Variables Ca, Mg, and K have a decisive influence on the component C_1_. In a similar way, components C_2_ and C_3_ can be related to the original variables. Consequently, this procedure allows analyzing the three-dimensional space, which was created based on the main components (Fig. 3).

**Fig. 4:**
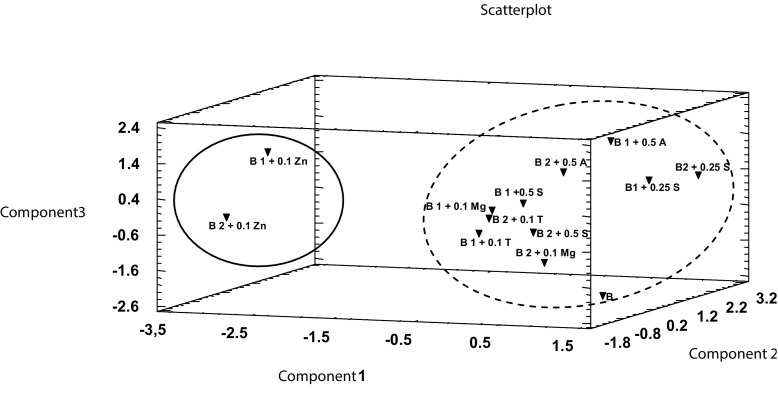
Main components C_1_, C_2_, and C_3_ in three-dimensional space representing the clusters of data. Shoot in vitro culture from MS medium (control) (*B*). In vitro culture from MS medium with addition of 0.1 g/L MgSO_4_ (*B 1 + 0.1 Mg* and *B 2 + 0.1 Mg*). In vitro culture from MS medium with addition of 0.1 g/L Zn hydroaspartate (*B 1 + 0.1 Zn* and *B 2 + 0.1 Zn*). In vitro culture from MS medium with addition of 0.1 g/L l-tryptophan (*B 1 + 0.1 T*; *B 2 + 0.1 T*). In vitro culture from MS medium with addition of 0.5 mg/L anthranilic acid (*B 1 + 0.5 A*; *B 2 + 0.5 A*). In vitro culture from MS medium with addition of 0.25 g/L serine (*B1 + 0.25 S*; *B2 + 0.25 S*). In vitro culture from MS medium with addition of 0.5 g/L serine (*B 1 + 0.5 S*; *B 1 + 0.5 S*). Data were presented as the mean ± SD; *n* = 4 repetitions. *p < 0.05, **p < 0.01 by Statistica 10 (StatSoft, Poland)

Based on the Fig. 4 which was created in three-dimensional space (principal components C_1_, C_2_, C_3_), two groups of objects can be clearly seen. The first of them is formed by in vitro cultures of *B. monnieri* grown on medium supplemented solely with the addition of 0.1 g/L Zn (solid line). The second cluster is formed by cultures enriched by the addition of 0.5 mg/L of anthranilic acid, 0.25 and 0.5 g/L of serine, 0.1 g/L of Mg, and 0.1 g/L of l-tryptophan (dashed line). Close position of the points indicates the presence of the similarity of the analyzed characteristics (concentration) within the analyzed group.

**Fig. 5:**
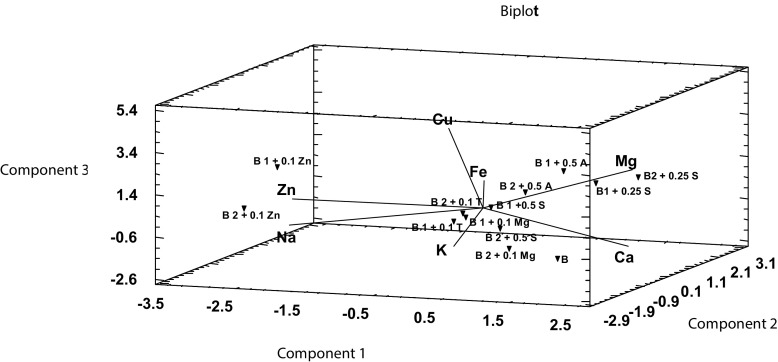
Biplot graph based on the main components C_1_, C_2_, and C_3_. Shoot in vitro culture from MS medium (control) (*B*). In vitro culture from MS medium with addition of 0.1 g/L MgSO_4_ (*B 1 + 0.1 Mg*, *B 2 + 0.1 Mg*). In vitro culture from MS medium with addition of 0.1 g/L Zn hydroaspartate (*B 1 + 0.1 Zn* and *B 2 + 0.1 Zn*). In vitro culture from MS medium with addition of 0.1 g/L l-tryptophan (*B 1 + 0.1 T*; *B 2 + 0.1 T*). In vitro culture from MS medium with addition of 0.5 mg/L anthranilic acid (*B 1 + 0.5 A*; *B 2 + 0.5 A*). In vitro culture from MS medium with addition of 0.25 g/L serine (*B1 + 0.25 S*; *B2 + 0.25 S*). In vitro culture from MS medium with addition of 0.5 g/L serine (*B 1 + 0.5 S*; *B 1 + 0.5 S*). Data were presented as the mean ± SD; *n* = 4 repetitions. *p < 0.05, **p < 0.01 by Statistica 10 (StatSoft, Poland)

Biplot diagram (Fig. 5) made on the basis of principal component analysis enables a clear and transparent tracking of changes in the concentration of the selected elements within the analyzed cultures.

After analysis of biplot graphs (Fig. 5), it can be indicated that the addition of anthranilic acid to the culture medium leads to increased bioaccumulation of Mg. In vitro culture of *B. monnieri* with 0.1 g/L Zn is characterized by a high content of this element and higher bioaccumulation of sodium. In vitro cultures enriched with 0.5 g/L serine have a high capacity for potassium accumulation, and culture enriched with anthranilic acid lead to the opposite effect.

## Conclusion

The results presented herein, for the first time, prove biochemical potential of *B. monnieri* in vitro cultures to accumulate bioelements.

The modification of in vitro culture media with a set of organic compounds and bioelements lead to effective accumulation of the elements in biomass. Observed effects are not direct ones. Modifications made definitely influenced the elements uptake mechanism which in turn enabled to achieve bioaccumulation of the desired elements at expected levels.

Already at the present stage of research, enriched types of *B. monnieri* in vitro cultures can be proposed as a rich potential biotechnological source of biologically active bioelements and a good model for further studies on optimization of production of these materials as a supplement of diet. This suggests that the next step should be to estimate the release and bioavailability of elements from in vitro cultured shoots of *B. monnieri*.
